# Comparisons of conventional in vitro fertilization versus intracytoplasmic sperm injection in women with thyroid autoimmunity and non-male factor infertility, a propensity score matching analysis

**DOI:** 10.1038/s41598-023-46085-0

**Published:** 2023-11-03

**Authors:** Yuchao Zhang, Yanli Liu, Chunyan Shen, Yichun Guan

**Affiliations:** Department of Reproductive Medicine, The Third Affiliated Hospital of Zhengzhou University, No. 7 Kangfuqian Street, Erqi, Zhengzhou, 450052 Henan China

**Keywords:** Endocrinology, Diagnosis, Health services

## Abstract

The aim of the study is to compare the outcomes between the insemination methods of conventional in vitro fertilization and intracytoplasmic sperm injection in infertile women with thyroid autoimmunity and non-male factor infertility. This was a retrospective cohort study which included women with thyroid autoimmunity and non-male factor infertility. Reproductive outcomes such as embryo development parameters and clinical outcomes were compared between the two groups. The propensity score matching was applied to balance the general characteristics with significant differences between the two groups. Generalized estimating equations were used to explore the impact of ICSI on the embryo development potential of the inseminated oocytes. Sensitivity analysis using E-values was used to account for unknown confounders. After 1:2 propensity score matching, the general characteristics were all comparable. The good cleavage embryo rate, blastocyst utilization rate, and good blastocyst rate were significantly lower in the intracytoplasmic sperm injection group than those in the conventional in vitro fertilization group. After controlling for the confounding factors, intracytoplasmic sperm injection was significantly negatively associated with development of usable blastocysts and good blastocysts, while showed no impact on fertilized oocytes, usable cleavage embryos and good cleavage embryos. Although limited by the limited sample size, there were comparable clinical and obstetrical outcomes between conventional in vitro fertilization and intracytoplasmic sperm injection groups. Intracytoplasmic sperm injection neither improved the embryo development potential nor increased the clinical pregnancy and live birth rates compared to conventional in vitro fertilization in the studied population. Prospective studies that randomly divide the studied population in two the two groups and compare the reproductive outcomes are warranted.

## Introduction

Intracytoplasmic sperm injection (ICSI), which is characterized by the injection of a single spermatozoon into the ooplasm, is the promising choice of the most effectiveness insemination methods for the couples with sever male factor infertility. The last two decades have witnessed the dramatic increase of ICSI usage in the fields of ART treatment, especially in Europe and in the USA^1^. Despite the lack of solid evidence-based recommendations, the majority of the increase lies in the couples with advanced maternal age, fewer oocytes, prior failed fertilization, unexplained infertility, and cryopreserved oocytes^2–7^. However, it should be noted that the process of injection not only bypasses natural selection process but also potentially damages the oocytes, and that this technology requires special training so that the minimum damage could be achieved^8,9^. Furthermore, the comparisons of obstetric and neonatal outcomes between pregnancies following conventional in vitro fertilization (cIVF) and ICSI have not been adequately studied. In China, ICSI were mainly offered to the couples with male-factor infertility, prior failed fertilization or low fertilization rate, with a proportion of 19.2% in all the IVF cycles^10^.

Weghofer et al. claimed that when the thyroid function was optimized, thyroid antibodies significantly impaired the embryo quality^11^. Furthermore, The meta-analysis performed by Busnelli et al. demonstrated that women with positive thyroid antibodies (thyroid autoimmunity, TAI) had detrimental impact (increased risk of miscarriage and decreased chance of live birth) on the course of pregnancy achieved through IVF/ICSI^12^. On the other hand, the evidence shows that thyroid antibodies are present in both serum and ovarian follicular of women with TAI^13,14^. It has been hypothesized that the zona pellucida, which surrounds oocytes and plays an important role in the process of routine fertilization, expresses similar antigens as the thyroid tissue does^15^. As a consequence, the zona pellucida could be a potential target for thyroid antibodies in the follicle fluid, which may be associated poor oocyte quality and fertilization potential by an antibody-mediated cytotoxic effect^16^. The insemination method of ICSI has thus been proposed to overcome the detrimental effect mediated by TAI^17^. Currently, whether ICSI could improve the ART outcomes remained unanswered. So, the aim of the study is to compare the outcomes between cIVF and ICSI in infertile women with TAI and non-male factor infertility.

## Methods and materials

This was a retrospective cohort study. Infertile couple visiting the Department of Reproductive Medicine, the Third Affiliated Hospital of Zhengzhou University from Jan 2020 to Sep 2022 were primarily included. The female partners were screened for thyroid function and ovarian reserve before further treatment. The inclusive criteria were listed as follows. (1) The women received ovarian stimulation protocols; (2) the oocytes were successfully picked up; (3) the insemination methods were either cIVF or ICSI; (4) the cycles were completed with autologous sperm and oocytes. According to the aim of the study, the couples with male-factor infertility and inseminated oocytes obtained from rescued ICSI were firstly excluded. If either partner was present with chromosomal or genetic abnormalities, the couples were also excluded. And according to the local policy, due to the reason that ICSI was seldom the preferential choice for the couples with non-male factor infertility in the first treatment cycles, we did not restrict the number of treatment cycles in this study. The study was performed in accordance with the Code of Ethics of the Declaration of Helsinki. Informed consent were waived form the patients due to the retrospective design.

### Ovarian stimulation protocols

Generally, the ovarian stimulation protocols consisted of gonadotropin releasing hormone agonist (GnRH-a) protocols, gonadotropin releasing hormone antagonist (GnRH-ant) protocols, and mild stimulation protocols. Specifically, for women with good ovarian reserve, the GnRH-a protocols were used. For the women with poor ovarian response (POR), polycystic ovary syndrome (PCOS), and failed GnRH-a treatment, the GnRH-ant protocols were used. For the POR women with other conditions, mild stimulation protocols were used. Whatever the protocols were, serum hormone levels such as LH, E2 and P were routinely tested during the stimulation course, along with the monitor of follicle development by transvaginal ultrasound. Either conventional trigger (human chorionic gonadotropin, hCG) or dual trigger (hCG + GnRH-a) was chosen for the final oocyte maturation.

Oocytes were then picked up 36 h after trigger. Both insemination methods were carried out by senior embryologists. For cIVF, the sperm was prepared to the concentration of (5–10) × 10^6^/ml by using the Markler plate. A total of 40–60 μl of sperm were added into the dish with partners' oocytes. Then the sperm and oocytes were cultured together for insemination in the incubator for either 4–6 h or overnight to remove the granular cells. For ICSI, the injection of single sperm into the oocyte was performed 38–40 h after trigger. The fertilization and embryo assessment were performed by the senior embryologists. The quality cleavage embryos and blastocysts were accessed according to the Istanbul consensus and described in previous studies^18–20^. Specifically, for cleavage embryos, quality was scored mainly based on the blastomere number, blastomere size, and fragmentation. Cleavage-stage embryos with good quality were defined as the number of even blastomere ranging from 6 to 10, and fragmentation being less than 20%. For blastocysts, quality was scored mainly based on the stage of development (3, 4, 5, 6), inner cell mass (A, B, C), and trophectoderm (A, B, C). Blastocysts with good quality were defined as the stage of development being higher than 3, and inner cell mass and trophectoderm both being better than C.

The women were transferred with either cleavage embryos or blastocyst in the fresh cycles. For cleavage-stage embryos, no more than two embryos were selected for transfer. For blastocyst, only one blastocyst was chosen for transfer. The transfer strategy was carried out according to the patient’s specific condition.

### Thyroid function test

The serum thyroid-stimulating hormone (TSH), free thyroxine (FT4), triiodothyronine levels (FT3), thyroid peroxidase antibodies (TPO-Ab), and thyroglobulin antibodies (Tg-Ab) were used for the assessment of thyroid function. The measurements were generally performed along with the basal sex hormone assessment. All the blood samples were collected on the 2nd–5th morning of the menstrual cycle. The samples were centrifuged at 3000 rpm for 10 min after half an hour of sampling. The serum on the upper layer was used for analysis. The measurements were all conducted by electrochemical luminescence (ECLIA) on a Cobas 8000 (Roche Diagnostics, Germany). The reference ranges of all the markers were provided by the assay. Daily internal quality control and yearly external quality control were carried out by request.

### Definition of fertility outcomes

The primary outcomes were the parameters that associated with embryo development potential. Fertilization rate was defined as the appearance of two pronucleus (2PN) per retrieved (inseminated/injected) oocytes; Cleavage embryo utilization rate was defined as number of cleavage (day 3) embryos which were available for transfer, cryopreservation, or culturing for blastocyst per fertilized oocytes; Good embryo rate was defined as usable cleavage embryos with good quality per fertilized oocytes; Blastocyst utilization rate was defined as appearance of blastocysts (day 5/6/7) which were available for transfer or cryopreservation per cultured cleavage embryos; Good blastocyst rate was defined as usable blastocysts with good quality per cultured cleavage embryos. The secondary outcomes were the implantation rate, clinical pregnancy rate, live birth rate, and miscarriage rate, which were described in previous studies^19,20^.

### Statistical analysis

Continuous variables were expressed as mean [standard deviation (SD)] or median [interquartile range (IQR)] based on the distribution of data, and categorical variables as number (percentage). Comparisons were performed by student-t test (normal distribution) or Mann–Whitney U-test (non-normal distribution) for continuous variables and chi-square analysis tests or Fishers exact test for categorical variables. Due to fact that ICSI was mainly offered to those with male factor infertility or failed IVF treatment, it was expected that apparent differences in terms of patients' general characteristics existed between cIVF group and ICSI group. In order to compare the outcomes between cIVF and ICSI groups with comparable general characteristics, the propensity score matching (PSM) analysis was used to balance the huge differences between the two groups. Embryos development parameters were compared after PSM. With the usage of data of each embryo’s outcomes, generalized estimating equations (GEE) was used to further explore the impact of ICSI on embryo development potential. E-values analysis were used to account for unknown confounders^21^. Data analyses were performed by SPSS (Version 22.0 IBM; NY). Significance was set at two-tailed P < 0.05 in all analyses.

### Ethical approval

The ethics committee of the Third Affiliated Hospital of Zhengzhou University approved this study.

### Informed consent

Informed consent was waived by the ethics committee of the Third Affiliated Hospital of Zhengzhou University due to the retrospective design.

## Results

For the 633 infertile women with TAI and non-male factor fertility, a total of 514 women were in the cIVF group, while 119 in the ICSI group. As shown in Table 1, there were significant differences between the two groups in terms of the couples age, proportion of ovarian stimulation protocols, and proportion of recurrent spontaneous abortion. (all P < 0.001). Although the number of AFC in the cIVF group was significantly higher than that in the ICSI group (P = 0.035), serum AMH levels did not show the statistical significance (P = 0.089). After 1:2 PSM, the general characteristics were all comparable between cIVF and ICSI groups.

**Table 1:** General characteristics of included patients between cIVF and ICSI.

	Before PSM			After PSM		
	IVF (514)	ICSI (119)	P	IVF (185)	ICSI (116)	P
Female age^a^	34 (5.3)	36 (5.6)	< 0.001	36 (5.3)	36 (5.6)	0.987
Male age^a^	34 (5.7)	36.4 (6.6)	< 0.001	36.2 (6.4)	36.5 (6.7)	0.763
AFC^a^	14.3 (8.1)	12.5 (7.9)	0.035	10.9 (7.7)	12.5 (8)	0.086
AMH^b^	16 (20.6)	12.4 (16.6)	0.089	8.6 (17.7)	12.2 (15.7)	0.072
FSH^b^	6.8 (3.1)	6.7 (3.1)	0.535	7.3 (4.2)	6.7 (3.1)	0.174
LH^b^	4.9 (3.4)	4.5 (2.4)	0.075	4.7 (3.1)	4.5 (2.4)	0.351
BMI^a^	23.6 (3.1)	23.8 (3)	0.652	23.7 (2.8)	23.9 (3.1)	0.505
Duration of infertility^b^	3 (3.5)	2.8 (3.5)	0.983	3 (4)	3 (3.5)	0.947
Type of infertility^c^						
Primary	151 (29.4)	28 (23.5)	0.202	30 (16.2)	28 (24.1)	0.090
Secondary	363 (70.6)	91 (76.5)		155 (83.8)	88 (75.9)	
Cause of infertility^c^						
Endometriosis	35 (6.8)	6 (5)	0.065	7 (3.8)	6 (5.2)	0.288
Ovulation dysfunction	41 (8)	9 (7.6)		9 (4.9)	9 (7.8)	
Decreased ovarian reserve	59 (11.5)	21 (17.6)		36 (19.5)	20 (17.2)	
Pelvic and fallopian tube factors	242 (47.1)	40 (33.6)		82 (44.3)	39 (33.6)	
Mixed factors	79 (15.4)	25 (21)		34 (18.4)	24 (20.7)	
Unexplained	58 (11.3)	18 (15.1)		17 (9.2)	18 (15.5)	
Ovarian stimulation protocols^c^						
GnRH-a	248 (48.2)	23 (19.3)	< 0.001	50 (27)	23 (19.8)	0.128
GnRH-ant	134 (26.1)	47 (39.5)		51 (27.6)	44 (37.9)	
Mild stimulation	132 (25.7)	49 (41.2)		84 (45.4)	49 (42.2)	
Thyroid function^c^						
Euthyroid	318 (61.9)	79 (66.4)	0.358	107 (57.8)	76 (65.5)	0.184
Thyroid dysfunction	196 (38.1)	40 (33.6)		78 (42.2)	40 (34.5)	
Recurrent spontaneous abortion^c^						
No	477 (92.8)	86 (72.3)	< 0.001	149 (80.5)	85 (73.3)	0.140
Yes	37 (7.2)	33 (27.7)		36 (19.5)	31 (26.7)	

Data were expressed as a: mean (SD); b: median (IQR); and c: n (%).

The number of oocytes picked up in the ICSI group were significantly higher than that in the cIVF group. However, the embryo development parameters were similar between the two groups (all P > 0.05). The 2PN rate per oocyte was expected to be significantly lower in the ICSI group due to the fact that the immature oocyte which would otherwise possibly become mature and capable of fertilization, were discarded after removing the granulosa cells. The good cleavage embryos rate, blastocyst utilization rate, and good blastocyst rate were significantly lower in the ICSI group than those in the cIVF group (P = 0.026, < 0.001, and < 0.001) (Table 2).

**Table 2:** Treatment outcomes of IVF versus ICSI in infertile women with positive thyroid antibodies after PSM.

	IVF (185)	ICSI (116)	P
Oocytes (n)	9 (8.3)	10 (9)	0.005
Fertilized (2PN) oocytes (n)	7.5 (7)	6 (5)	0.883
Usable embryos (n)	7 (6)	5 (5.8)	0.858
Good embryos (n)	6 (5)	4 (4)	0.344
Usable blastocyst (n)	4 (4)	2 (3)	0.813
Good blastocyst (n)	2 (3.3)	1.5 (3)	0.214
2PN rate per oocytes (%)	985 (73%)	611 (56.4%)	< 0.001
2PN rate inseminated or injected (%)	985 (73%)	611 (76.6%)	0.065
Cleavage embryo utilization rate (%)	805 (83.9%)	482 (80.2%)	0.065
Good embryos rate (%)	505 (52.6%)	282 (46.9%)	0.029
Blastocyst utilization rate (%)	315 (59.1%)	181 (46.9%)	< 0.001
Good blastocyst rate (%)	146 (27.4%)	56 (14.5%)	< 0.001

After adjusted for female age, BMI, AFC number, ovarian stimulation protocols, miscarriage history, and type of fertility, The insemination method of ICSI showed non-significant impact on normal fertilized oocytes (adjusted OR = 0.98, P = 0.917), usable cleavage embryos (adjusted OR = 0.71, P = 0.097) and good cleavage embryo (adjusted OR = 0.79, P = 0.131). However, ICSI seemed to negatively impact the development of usable blastocyst (adjusted OR = 0.54, P = 0.002) and good blastocyst (adjusted OR = 0.39, P < 0.001). The adjusted E-values for usable and good blastocysts were 2.77 and 3.20 with the upper limit of the confidence interval being 2.70 and 2.63, respectively (Table 3).

**Table 3:** Impact of ICSI versus IVF on the embryo development potential in women with positive thyroid antibodies after PSM.

	OR (95%CI)	P	E-value (RR)	E-value (UCL)	Adjusted OR (95%CI)	P	E-value (RR)	E-value (UCL)
2PN rate per oocyte	1.04 (0.75, 1.45)	0.798	2.02	1.00	0.98 (0.33, 1.37)	0.917	2.02	1.00
Embryo utilization rate	0.72 (0.48, 1.06)	0.096	2.36	1.00	0.71 (0.47, 1.07)	0.097	2.37	1.00
Good embryos rate	0.79 (0.68, 1.08)	0.132	2.25	1.00	0.79 (0.58, 1.07)	0.131	2.25	1.00
Blastocyst utilization rate	0.58 (0.42, 0.79)	0.002	2.63	2.53	0.54 (0.39, 0.74)	0.003	2.77	2.70
Good blastocyst rate	0.44 (0.28, 0.70)	< 0.001	3.02	2.86	0.39 (0.26, 0.58)	< 0.001	3.20	2.63

E-values were calculated based on the study performed by Tyler J. VanderWeele^21^.

*RR* relative risk, *OR* odd ratio, *UCL* upper lower 95% confidence limit.

Finally, the clinical outcomes between cIVF and ICSI groups were also compared. The female age, AMH levels, and total AFC number, and transfer embryos were similar between the two groups. The implantation rate, clinical pregnancy rate and live birth rate were slightly higher in the cIVF group without statistic significance. The miscarriage rate in the cIVF group was 26.8%, while in the ICSI group, no miscarriage was observed. Furthermore, the gestational weeks, and proportions of preterm birth and cesarean section were also comparable between the two groups (Table 4).

**Table 4:** Clinical outcomes of IVF versus ICSI in women with positive thyroid antibodies after PSM.

	IVF (81)	ICSI (22)	P
Female age	34.4 ± 4.5	33.6 ± 5.1	0.488
AMH	20.2 ± 19.2	23.2 ± 17.7	0.521
AFC	13.8 ± 6.7	16.1 ± 7.6	0.163
Transferred embryos			
Cleavage embryos	70.4 (57/81)	77.3 (17/22)	0.318
Blastocyst	29.6 (24/81)	22.7 (5/22)	
Implantation rate	38.6 (49/127)	25.7 (9/35)	0.160
Clinical pregnancy rate	50.6 (41/81)	31.8 (7/22)	0.117
Live birth rate	37.0 (30/81)	31.8 (7/22)	0.651
Miscarriage rate	26.8 (11/41)	0 (0/7)	0.170
Singleton	76.7 (23/30)	71.4 (2/7)	1.000
Gestational weeks	36.8 ± 2.7	37.3 ± 2.3	0.665
Preterm birth	26.7 (8/30)	42.9 (3/7)	0.403
Cesarean section	76.7 (23/30)	85.7 (6/7)	1.000

## Discussion

In this retrospective study, we included infertile women with TAI and non-male infertility, and compared the ART outcomes between the insemination methods of cIVF and ICSI. The results showed that ICSI did not increased the embryo development potential and clinical outcomes compared to cIVF. Although miscarriage was not present in the ICSI group, we tend to believed it was the limited sample size that led to a false result.

The associations between TAI and reproductive outcomes had long been investigated. However, the conclusions remained debatable. Regardless of the newly published studies claiming that no detrimental effects of thyroid antibodies on reproductive outcomes in women undergoing ART treatment, there were also evidences showing that TAI were associated with not only low fertilization rate and number of good quality embryo, but also increased risks of pregnancy loss and preterm birth^22–25^. Rao et al. demonstrated that thyroid antibodies in euthyroid women were neither associated with embryo quality nor cumulative live birth rate^26^. However, in this study, we demonstrated that thyroid antibodies showed impaired effect on embryo development (Supp Table 1).

Monteleone et al. demonstrated that in women with TAI, it was the those offered with insemination of ICSI instead of cIVF that were pregnant^27^. The meta-analysis performed by Poppe et al. included women who were with and without TAI and undergoing ICSI treatment, and showed comparable miscarriage rates, which suggested that ICSI may overcome the impeding effects of thyroid antibodies on oocytes and embryos^22^. Although it had been well accepted that ICSI was able to help the couples with sever male infertility successfully get pregnant, its value for couple with non-male factor infertility had been questioned by many researchers^2,28,29^. Tan et al. demonstrated that thyroid antibodies per se do not impair ICSI outcome in euthyroid healthy women. However, comparisons between ICSI and cIVF were lacking, and only couples with male factors were included^30^. The most consistent conclusions were that ICSI neither improved the embryo quality nor resulted in a higher (cumulative) live birth rate compared with IVF for those couples^3,31–35^. Therefore, it was also reasonable to challenge the value of ICSI used for women with TAI and non-male factor infertility. To answer this question, we strictly included women of interest, and offered them with either cIVF or ICSI. However, significant difference in terms of the general characteristics of women between the insemination methods of cIVF and ICSI were observed, which would have extensively lowered the reliability of the study if reproductive outcomes were directly compared. As a consequence, PSM was applied to balance the general characteristics between the two groups. In such condition, we demonstrated that ICSI reduced the usable (good) blastocyst rates instead of improving the embryo quality for this population, and that the impaired impact of ICSI remained after controlling for female age, cause of infertility, thyroid function. The conflicting results may be explained by the studied population (TAI and non-male factor infertility), retrospective study design, and strict application of ICSI in our country. On the other hand, although comparable general characteristics were observed between the groups of ICSI and cIVF after PSM, the women in the ICSI group had a slightly higher AMH levels and AFC numbers, which were strongly associated with ovarian reserve.

Furthermore, the clinical outcomes were also compared between ICSI and cIVF in women with TAI, which were not mentioned in previous studies. The results were consistent with previous studies that ICSI did not improve the clinical outcomes compared to cIVF for couples with non-male factor infertility^33,35,36^. However, it was interesting to find that no women in the ICSI group ended up with miscarriage. This was an interesting results, because the miscarriage rates for the general population were comparable between the insemination methods of IVF and ICSI (13.2% vs 13.5%) in our department. As a matter of fact, we tend to believed that the limited sample size played an important role in misleading the results.

There were some limitations in this study. First of all, the sample size was relatively small due to the low proportion of the studied population, which may reduce the power of statistic analysis. Secondly, this was a retrospective study which may lead to selection bias. However, we performed the PSM analysis which may overcome the discrepancy of general characteristics between the two groups. Indeed, a prospective study which randomly divide the women into cIVF group or ICSI group could definitively provide solid data about the impact of ICSI on the reproductive outcomes in women with TAI and non-male factor infertility. Thirdly, although couples with male-factor infertility were excluded in this study, there were couples who were offered with ICSI, which implied that the total number of prepared sperm were not enough for cIVF insemination, which indicated a risk of male factor problems, or that the couples may suffer with failed ART treatment in other hospitals.

The strengths of the study were listed as follow. Firstly, although previous studies investigated the role of ICSI in couple with non-male infertility, this study further narrowed the population with positive thyroid antibodies. As far as we know, this was the first study exploring the reproductive outcomes between cIVF and ICSI groups in the study population. Secondly, analysis based on each embryo’s outcomes were performed by GEE, which may further illustrate the impact of ICSI compared to cIVF Thirdly, although limited by the sample size, both short-term reproductive outcomes such as embryo quality and long-term reproductive outcomes such as clinical pregnancy rate and live birth rate were investigated, which may add further understanding of the role of ICSI in the studied population.

## Conclusion

Even though no miscarriage were observed in women with insemination method of ICSI, the result was mostly believed to be caused by the limited sample size. In total, ICSI neither improved the embryo development potential nor increased the clinical pregnancy and live birth rates compared to cIVF in women with TAI and non-male factor infertility. Prospective studies that randomly divide the studied population in two the two groups and compare the reproductive outcomes are warranted.

### Supplementary Information

Supplementary Table 1.

## Data Availability

The data used during the current study are available from the corresponding author on a reasonable request.
