# Supplementary material for: Comparisons of conventional in vitro fertilization versus intracytoplasmic sperm injection in women with thyroid autoimmunity and non-male factor infertility, a propensity score matching analysis
Source: Sci Rep. 2023 Nov 3;13:18967. doi: 10.1038/s41598-023-46085-0 (PMC10624835; doi:10.1038/s41598-023-46085-0)
Supplement: Supplementary file 1 — Supplementary Table 1. [file 41598_2023_46085_MOESM1_ESM.docx]

Supplementary Table 1 Treatment outcomes in infertile women with positive VS negative thyroid antibodies

|  | Positive thyroid antibodies(633) | Negative thyroid antibodies(3257) | P |
| --- | --- | --- | --- |
| Oocytes (n) | 9.0(10.0) | 9.0(11.0) | 0.581 |
| Fertilized (2PN) oocytes (n) | 6.0(7.0) | 6.0(9.0) | 0.465 |
| Available embryos (n) | 4.0(6.0) | 5.0(7.00) | 0.351 |
| Good embryos (n) | 3.0(4.0) | 3.0(5.0) | 0.410 |
| Available blastocyst (n) | 1.0(4.0) | 2.0(5.0) | 0.142 |
| Good blastocyst (n) | 0(2.0) | 0(2.0) | 0.226 |
| 2PN rate per oocytes (%) | 4408(69.3%) | 23971(71.5%) | 0.001 |
| Available embryos rate (%) | 3666(85.0%) | 20140(86.0%) | 0.065 |
| Good embryos rate (%) | 2311(53.6%) | 12955(55.3%) | 0.031 |
| Available blastocyst rate (%) | 1652(59.1%) | 9624(61.2%) | 0.043 |
| Good blastocyst rate (%) | 676(24.2%) | 3795(24.1%) | 0.928 |
